# Epithelial‐mesenchymal transition may be involved in the immune evasion of circulating gastric tumor cells via downregulation of ULBP1

**DOI:** 10.1002/cam4.2871

**Published:** 2020-02-20

**Authors:** Baoguang Hu, Xiaokun Tian, Yanbin Li, Yangchun Liu, Tao Yang, Zhaodong Han, Jiajia An, Lingqun Kong, Yuming Li

**Affiliations:** ^1^ Department of Gastrointestinal Surgery Binzhou Medical University Hospital Binzhou China; ^2^ Department of Burn and Plastic Surgery the Sixth People's Hospital of Zibo Zibo China; ^3^ Jiangxi Medical College Queen Mary College of Nanchang University Nanchang China; ^4^ Department of Clinical Laboratory Binzhou Medical University Hospital Binzhou China; ^5^ Department of Hepatobiliary Surgery Binzhou Medical University Hospital Binzhou China

**Keywords:** circulating tumor cells, epithelial‐mesenchymal transition, gastric cancer, immune evasion, transforming growth factor beta 1

## Abstract

**Background:**

Increasing numbers of studies have demonstrated that circulating tumor cells (CTCs) undergo a phenotypic change termed epithelial‐mesenchymal transition (EMT), and researchers have proposed that EMT might provide CTCs with increased potential to survive in the different microenvironments encountered during metastasis through various ways, such as by increasing cell survival and early colonization. However, the exact role of EMT in CTCs remains unclear.

**Methods:**

In this study, we identified CTCs of 41 patients with gastric cancer using Cyttel‐CTC and im‐FISH (immune‐fluorescence in situ hybridization) methods, and tested the expression of EMT markers and ULBP1 (a major member of the NKG2D—natural killer [NK] group 2 member D—ligand family) on CTCs. Moreover, we investigated the relationship between the expression of EMT markers and ULBP1 on CTCs and gastric cancer cell lines.

**Results:**

Our results showed that the CTCs of gastric cancer patients exhibited three EMT marker subtypes, and that the expression of ULBP1 was significantly lower on mesenchymal phenotypic CTCs (M^+^CTCs) than on epithelial phenotypic CTCs (E^+^CTCs). EMT induced by TGF‐β in vitro produced a similar phenomenon, and we therefore proposed that EMT might be involved in the immune evasion of CTCs from NK cells by altering the expression of ULBP1.

**Conclusions:**

Our study indicated that EMT might play a vital role in the immune invasion of CTCs by regulating the expression of ULBP1 on CTCs. These findings could provide potential strategies for targeting the immune evasion capacity of CTCs.

## Graphical abstract

In this proof‐of‐principle study, we demonstrated that gastric CTCs in peripheral blood exhibited obvious EMT characteristics, as well as a downregulation of NKG2D ligands, with TGF‐β1‐induced EMT accompanied by this downregulation of NKG2D ligands in vitro. Considering these results, we conclude that EMT of gastric CTCs might be involved in the immune evasion of CTCs through shedding or restricting the expression of NKG2D ligands during the process of metastasis.

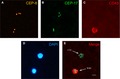

## 1 INTRODUCTION

Gastric cancer is among the most common malignant digestive system tumors worldwide.^1^ Despite advances in the prevention, detection, and adjuvant treatment of gastric cancer, the number of people diagnosed with gastric cancer is increasing, and metastasis remains a major causative factor of cancer‐related death.^2^, ^3^, ^4^, ^5^ However, the mechanism underlying metastasis remains unclear. Recently, studies have demonstrated that metastasis is a multi‐step process that involves the presence of circulating tumor cells (CTCs) in the bloodstream and their growth in other organs.^6^, ^7^, ^8^ CTCs are singular cell or small cell masses that disseminate from primary tumors into blood vessels, and are usually identified by the following criteria: (a) atypical phenotype, with high nuclear to cytoplasm ratio or irregular shape; (b) intratumor heterogeneity, including the amplification of aneuploidy in chromosomes, altered cell‐surface antigen markers, molecular diversity, and varying mobility potential.^9^, ^10^, ^11^ Since the first reports on CTCs, the enumeration and analysis of the cells have attracted increasing attention due to their crucial roles in the metastatic cascade and the progression of metastasis. At present, CTC detection, via a novel method termed “liquid biopsies,” is widely used in the clinical treatment of various malignant tumor types.^12^, ^13^, ^14^, ^15^ Compared to traditional tissue biopsy of solid neoplasms, noninvasive liquid biopsies are considered to be superior, and enable clinicians to use blood samples of only a few milliliters from cancer patients to perform "real‐time" monitoring and analysis of the cancer, to obtain more information at an earlier stage.^16^, ^17^, ^18^ Recently, studies have revealed that CTCs of gastric cancer move from the primary tumor into the bloodstream and travel to other organs during the early stage, thus exerting a dominant contribution to early metastasis.^19^, ^20^ In this process, CTCs undergo a phenotypic change termed epithelial‐mesenchymal transition (EMT), in which the cells acquire a mesenchymal phenotype and thus become more aggressive and invasive.^21^, ^22^, ^23^ Moreover, researchers have proposed that EMT might provide CTCs with increased potential to survive in the different microenvironments encountered during metastasis through various ways, such as by enhancing metastatic competence, by increasing cell survival and early colonization, by promoting the formation of specific protective cytoskeletal structures and clusters of CTCs, and by promoting immune evasion.^24^, ^25^, ^26^, ^27^ However, despite extensive experimental data, the exact role of EMT remains unclear, particularly regarding CTC immune evasion in the peripheral bloodstream.

The present study aimed to explore the role of EMT in the immune escape of gastric CTCs. We used Cyttel‐CTC and im‐FISH (immune‐fluorescence in situ hybridization) methods to detect CTCs and to investigate the expression of EMT markers and ULBP1 on CTCs.^28^, ^29^, ^30^ Moreover, we investigated the relationship between the expression of EMT markers and ULBP1 in vitro in gastric cancer cell lines. Our results suggested that the expression of ULBP1 was significant lower on mesenchymal phenotypic CTCs than on epithelial phenotypic CTCs, and that EMT induced by TGF‐β in vitro could produce a similar change. We therefore proposed that EMT might be involved in the immune escape of CTCs from natural killer (NK) cell killing through altered expression of ULBP1 on the tumor cells. To the best of our knowledge, this is the first study to analyze the EMT phenomenon combined with ULBP1 expression status in circulating gastric carcinoma cells.

## 2 MATERIAL AND METHODS

### 2.1 Patients and sample collection

A total of 41 patients with newly diagnosed gastric cancer from the Department of Gastrointestinal Surgery of Binzhou Medical University Hospital (Binzhou City, China) were included in the study from July to September 2016. The diagnosis was confirmed by histopathological analysis and the patients did not receive any treatments, such as neoadjuvant chemical therapy or radiotherapy, prior to sample collection. 3.2‐mL peripheral blood samples were collected using 5‐mL tubes with ACD anti‐coagulant (BD Technology; 0.8 mL) after discarding the first 2 mL to avoid potential skin cell contamination from the venipuncture. The blood was stored at room temperature for no more than 24 hours for further analysis. Clinical information on the patients, including age and gender, was collected. The Medical Ethical Committee of the Affiliated Hospital of Binzhou Medical University approved this research, and written informed consent was obtained from the patients prior to blood collection.

### 2.2 Enrichment of CTCs in peripheral blood

A technique based on karyotypic in situ characterization of CTCs was used to detect CTCs in peripheral blood. The procedure was similar to that reported previously by Ge et al.^31^ In brief, 3.2‐mL blood samples were collected in 5‐mL tubes, then transferred into 50‐mL tubes containing 40 mL CS1 working solution (Cyttel) after thorough mixing. Subsequently, the solution was centrifuged at 500× g for 5 minutes at room temperature. Supernatants were discarded and CS2 working buffer (Cyttel) was added into the tubes to remove the red blood cells. CS3 working buffer (Cyttel) and magnetic beads (Cyttel) were subsequently used to deplete the majority (>99.9%) of leukocytes by magnetic separation and gradient centrifugation. The middle cell layer containing rare cells was transferred to another tube for further centrifugation. Following centrifugation, the upper liquid (100 µL) was discarded and 200 µL CF1 stationary liquid (Cyttel) was added to resuspend the remaining cells. Finally, the cells were applied onto coated CTC PEN membrane slides and dried overnight at room temperature for immediate use or storage at −20°C.

### 2.3 Combined immunocytochemistry staining and FISH

After the dry slides were dehydrated in an ethanol series (75%, 85%, and 100%; 3 minutes each), the slides were incubated with a cocktail of Alexa Fluor 594‐conjugated anti‐CD45 (Cytelligen) for 1 hour in the dark. Subsequently, FISH was performed with centromere probe 8 (orange fluorescent) (Abbott Molecular Diagnostics) and 17 (green fluorescent) (Abbott Molecular Diagnostics) using an s500 Statspin ThermoBrite Slide Hybridization/Denaturation System (Abbott Molecular) for 1.5 hours at 37°C. Finally, the slides were mounted with mounting media containing 4'‐6‐diamidino‐2‐phenylindole (DAPI) (Life Technology) and analyzed with an automatic fluorescent microscope using a 40× objective lens (Olympus BX53). Genomic aberrations and negative expression of CD45 are hallmarks of malignant cells, and CTCs were identified as CEP8^+^/CD45^−^/DAPI^+^ or CEP17^+^/CD45^−^/DAPI^+^.

### 2.4 Detection of Vimentin, EpCAM, and ULBP1 surface expression on CTCs

The identified CTCs were isolated using a laser capture microdissection system‐Leica LMD6500 (Leica). After fixation and dehydration, the CTCs were incubated with a cocktail of Vimentin RNA probe‐labeled AlexaFluor594 (Invitrogen), EpCAM RNA probe‐labeled AlexaFluor488 (Invitrogen) and ULBP1 RNA probe‐labeled AlexaFluor647 (Invitrogen) (capture probe sequences are shown in table 4) in the s500 Statspin ThermoBrite Slide Hybridization/Denaturation System for 1.5 hours at 37˚C. Subsequently, the CTCs were washed three times with phosphate‐buffered saline (PBS), then mounted with DAPI (Life Technology) containing mounting media, and subjected to image analysis using a three‐color fluorescence microscope.

### 2.5 Cell culture and EMT induction

The human gastric carcinoma cell line SGC7901 was obtained from the Center Laboratory of Binzhou Medical University. Cells were cultured in RPMI 1640 medium (Thermo Fisher Scientific, Inc) supplemented with 10% fetal bovine serum (Thermo Fisher Scientific, Inc) and 2% penicillin/streptomycin in a humidified atmosphere with 95% air and 5% CO_2_, in an incubator (Thermo Fisher Scientific, Inc) at 37°C. EMT was induced via the application of exogenous recombinant human TGF‐β1 (Thermo Fisher Scientific, Inc) at concentrations of 5, 10, and 20 ng/mL. The cells were collected for subsequent analysis at 72 hours after the addition of TGF‐β1.

### 2.6 Western blot analysis

After the total protein was extracted and quantitated using a BCA Protein Assay Kit (Solarbio), equal amounts of protein (30‐50 μg per lane) were subjected to 10% sodium dodecyl sulfate‐tris glycine polyacrylamide gel electrophoresis (Solarbio), then electroblotted on to a polyvinylidene fluoride membrane (Solarbio). After blocking in 5% nonfat milk (BD Technology), the membrane was incubated with primary antibodies against EpCAM, Vimentin, MICA, and ULBP1/2 (Abcam Technology) at dilutions of 1:1000‐2000 overnight, then with horseradish peroxidase (HRP)‐conjugated goat anti‐mouse secondary antibody (ZSGB‐BIO) at a dilution of 1:10 000‐20 000. Immunocomplexes were visualized using an enhanced chemiluminescence detection system (Billerica, MA, USA). GAPDH (GOOD HERE Technology) was used as an internal reference for comparison with the other proteins.

### 2.7 Immunofluorescence

Following treatment with 20 ng/mL TGF‐β1 for 72 hours, cultured SGC7901 cells on glass chamber slides (Thermo Fisher Scientific, Inc) were fixed with 4% paraformaldehyde in PBS (Beyotime) for 15 minutes at room temperature, and then were washed three times with PBS. Subsequently, the fixed cells were permeabilized with 0.5% Triton X‐100 (Solarbio) in PBS for 10 minutes, then blocked with 1% BSA in PBS for 1 hour. Following this, the cells were incubated with a cocktail of primary antibodies against EpCAM, Vimentin, MICA, and ULBP1 (Abcam Technology) overnight at 4°C. The cells were then washed with PBS and incubated with Dylight 488/549‐conjugated secondary antibody for 1 hour at room temperature. Finally, the slides were mounted with mounting media containing DAPI (Life Technology) and analyzed with an automatic fluorescent microscope using a 40× objective lens (Olympus BX53).

### 2.8 Statistical analysis

Statistical analyses were performed using Statistical Package for Social Science Statistics 22.0 software (SPSS; IBM) and GraphPad Prism Software version 5.0 (San Diego, CA). The chi‐square test or Fisher's exact test were used to assess associations between CTC positivity and clinicopathological characteristics of the patients. One‐way ANOVA was used to evaluate the differences among groups. A value of *P* < .05 was considered to indicate a statistical significance.

## 3 RESULTS

### 3.1 Patient characteristics and detected gastric CTCs

**Figure 1:**
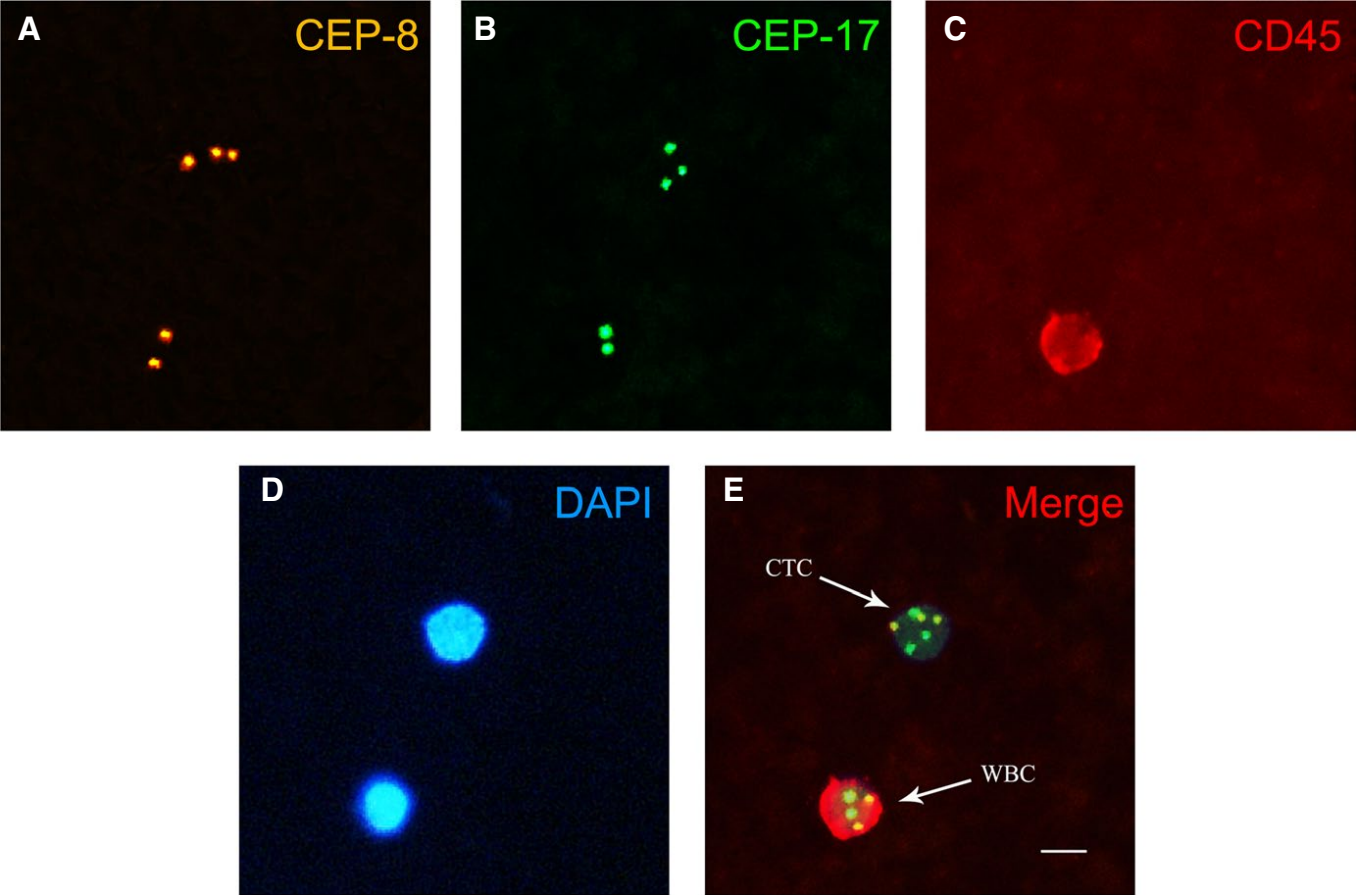
Detected circulating tumor cells (CTCs) in the peripheral blood of gastric cancer patients. A, CEP‐8 probe signal staining (yellow) in CTCs (three points) and white blood cells (WBCs) (two points). B, CEP‐17 probe signal staining (green) in CTCs (three points) and WBCs (two points). C, CD45 staining (red) in CTCs (negative) and WBCs (positive). D, DAPI staining (blue) in CTCs and WBCs. E, Merged image with probe signals and CD45 and nuclear staining; CTCs were identified as polyploid CEP8^+^/CD45^−^/DAPI^+^ or polyploid CEP17^+^/CD45^−^/DAPI^+^ cells, and WBCs were identified as diploid CEP8^+^/CD45^+^/DAPI^+^ or diploid CEP17^+^/CD45^+^/DAPI^+^ cells. Scale bar = 10 µm

As shown in Table 1, a total of 41 gastric cancer patients were included in this study: 30 males and 11 females, with a mean age of 64.37 ± 8.75 and 63.00 ± 7.27, respectively. CTCs were detected in 29 of the 41 patients (70.7%), and the CTC counts ranged from 0 to 13 cells/3.2 mL peripheral blood. As shown in Figure 1, CTCs were identified as polyploid CEP8^+^/CD45^−^/DAPI^+^ or polyploid CEP17^+^/CD45^−^/DAPI^+^ cells, while white blood cells (WBCs) were identified as diploid CEP8^+^/CD45^+^/DAPI^+^ and diploid CEP17^+^/CD45^+^/DAPI^+^ cells. We defined a CTC number of ≥2/3.2 mL peripheral blood as positive detection.

**Table 1:** Detected CTCs in 41 gastric cancer patients

Patients	Gender	Age	CTCs count (n)
1	F	48	3
2	M	68	5
3	M	68	4
4	M	44	4
5	M	65	13
6	M	66	2
7	M	62	2
8	M	74	0
9	M	57	2
10	M	51	3
11	M	78	0
12	M	80	2
13	M	61	0
14	M	50	3
15	F	73	0
16	M	70	12
17	F	72	1
18	M	64	8
19	F	64	6
20	M	72	1
21	M	61	6
22	M	62	9
23	F	62	6
24	M	65	12
25	M	50	2
26	M	58	1
27	M	82	3
28	M	67	0
29	F	64	2
30	F	62	8
31	F	64	0
32	M	68	2
33	M	60	6
34	M	71	4
35	F	53	0
36	M	66	0
37	F	64	1
38	M	69	7
39	M	62	4
40	F	67	4
41	M	60	4

Abbreviations: CTCs, circulating tumor cells; F, female; M, male; n, number.

### 3.2 EMT markers expressed on gastric CTCs

**Figure 2:**
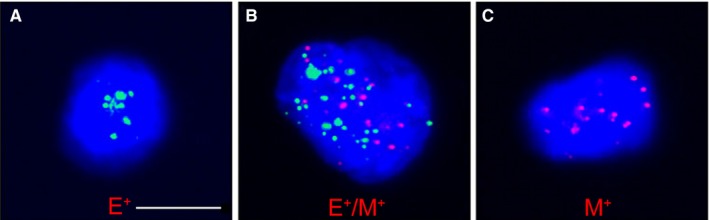
Three epithelial‐mesenchymal transition subtypes of circulating tumor cells (CTCs) in gastric cancer patients. Epithelial biomarkers (EpCAM) are indicated by green dots; mesenchymal biomarkers (Vimentin) are indicated by red dots. Cells with green dots represent epithelial CTCs (A), cells with red dots represent mesenchymal CTCs (C), and cells with red and green dots represent biphenotypic CTCs (B). Scale bar = 10 µm

To evaluate EMT phenotype in CTCs, we assessed the expression of EMT markers, including EpCAM and Vimentin, on the identified CTCs. As shown in Figure 2, we classified CTCs into three subpopulations based on the expression of EMT markers: Epithelial CTCs (Figure 2A, E^+^CTC), mesenchymal CTCs (Figure 2C, M^+^CTC) and biphenotypic (Figure 2B, E^+^/M^+^CTC) CTCs. These three types of CTCs exhibited high‐level expression of EpCAM, Vimentin, and both EpCAM and Vimentin, respectively. As shown in Table 2, E^+^CTCs were the most commonly identified subtype, with a positive rate of 93.1%, and were detected in the majority of CTC‐positive gastric patients. The second most common subtype was E^+^/M^+^CTC, with a positive rate of 86.21%. M^+^CTCs were identified in 22 of the 29 CTC‐positive patients, with a positive rate of 75.86%. The count ranges of the three CTC subpopulations were 0‐6, 0‐5, and 0‐4 cells/3.2 mL peripheral blood, respectively. Notably, the patients that presented with higher CTC counts seemed to exhibit higher counts of EMT‐phenotypic CTCs.

**Table 2:** Counts of EMT phenotypic CTCs in CTCs positive patients

Patients	Gender	Age	CTCs count (n)	E^+^CTCs count (n)	E^+^/M^+^CTCs count (n)	M^+^CTCs count (n)
1	M	66	2	1	1	0
2	M	62	2	2	0	0
3	M	57	2	1	1	0
4	M	80	2	2	0	0
5	M	50	2	1	1	0
6	F	64	2	1	0	1
7	M	68	2	0	0	2
8	F	48	3	1	2	0
9	M	51	3	2	1	0
10	M	50	3	1	1	1
11	M	82	3	0	2	1
12	M	68	4	1	2	1
13	M	44	4	1	1	2
14	M	71	4	2	1	1
15	M	62	4	1	2	1
16	F	67	4	2	1	1
17	M	60	4	1	1	2
18	M	68	5	2	1	2
19	F	64	6	3	1	2
20	M	61	6	2	3	1
21	F	62	6	2	2	2
22	M	60	6	1	3	2
23	M	69	7	2	2	3
24	M	64	8	4	2	2
25	F	62	8	3	3	2
26	M	62	9	4	2	3
27	M	70	12	3	5	4
28	M	65	12	4	5	3
29	M	65	13	6	4	3

Abbreviations: CTCs, circulating tumor cells; E^+^/M^+^CTCs, biophenotypic CTCs; E^+^CTCs, epithelial CTCs; EMT, epithelial‐mesenchymal transition; F, female; M, male; M^+^CTCs, mesenchymal CTCs.

### 3.3 ULBP1 expression was downregulated on E^+^/M^+^ and M^+^ CTCs

**Figure 3:**
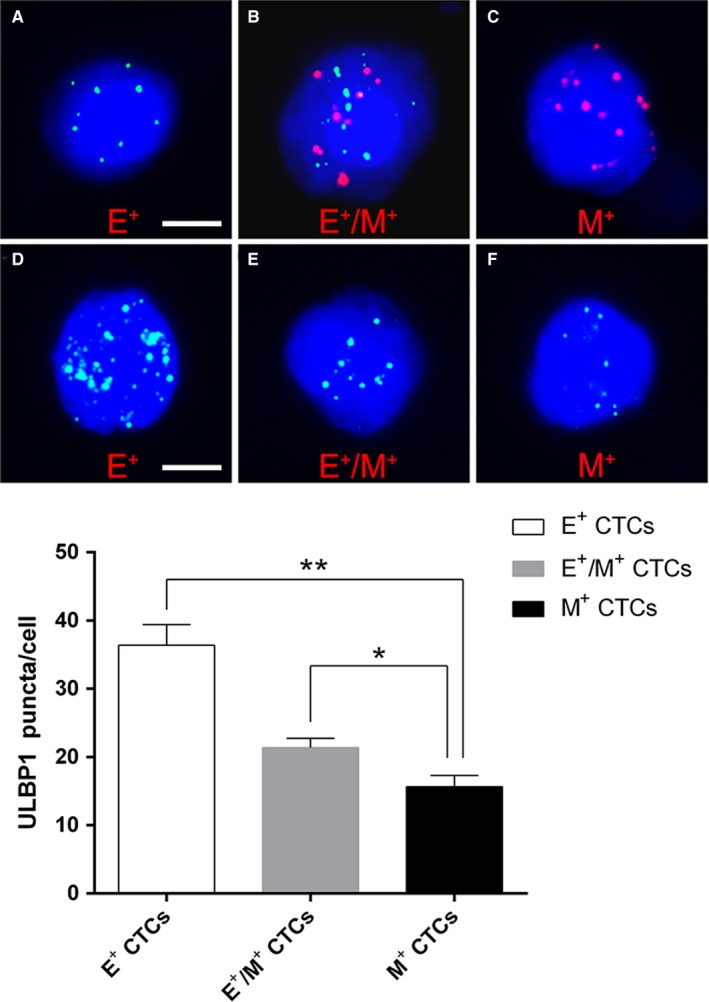
Surface expression of ULBP1 on circulating tumor cells (CTCs) with different epithelial‐mesenchymal transition (EMT) phenotypes. A‐C, Expression of EpCAM and Vimentin on predetermined CTCs (blue dots: epithelial biomarker expression; red dots: mesenchymal biomarker expression; scale bars = 5μm). D‐F, ULBP1 expression on different EMT‐phenotypic CTCs (bright blue dots: ULBP1 expression; scale bar = 10 μm). *P < .05, **P < .01

It is established that NKG2D ligands play a vital role in immune responses mediated by NK cells and CTLs, and that ULBP1 is a major member of the NKG2D ligand family. To further investigate the role of EMT in the immune evasion capacity of CTCs, we detected the expression of ULBP1 on the identified CTCs. For each of the different CTC subpopulations exhibiting the EMT phenotype, there were significant differences in the expression levels of ULBP1. Notably, the expression level of ULBP1 was substantially lower in the M^+^CTC subtype compared with the E^+^/M^+^ CTC and E^+^ CTC subtypes (Figure 3).

### 3.4 TGF‐β1‐induced EMT attenuates the expression of ULBP1 in gastric SGC7901 cells

**Figure 4:**
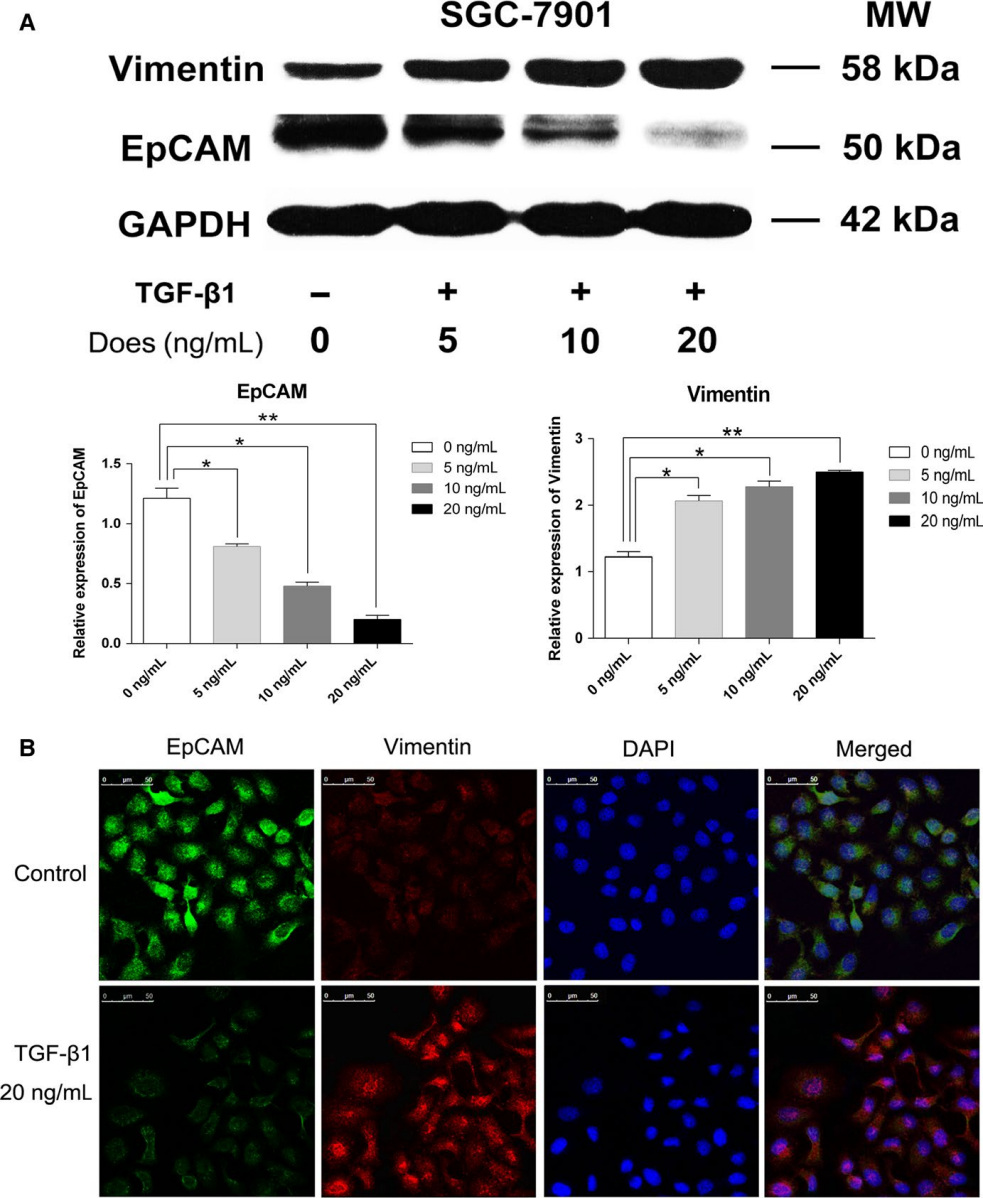
Epithelial‐mesenchymal transition (EMT) induced by TGF‐β1 in gastric SGC7901 cells. A, EpCAM and Vimentin expression in SGC7901 cells treated with TGF‐β1 at concentrations of 5, 10, and 20 ng/mL for 72 h. B, EMT induced by TGF‐β1 (20 ng/mL) in SGC7901 cells treated for 72 h. Scale bar = 50 μm. *P < .05, **P < .01

**Figure 5:**
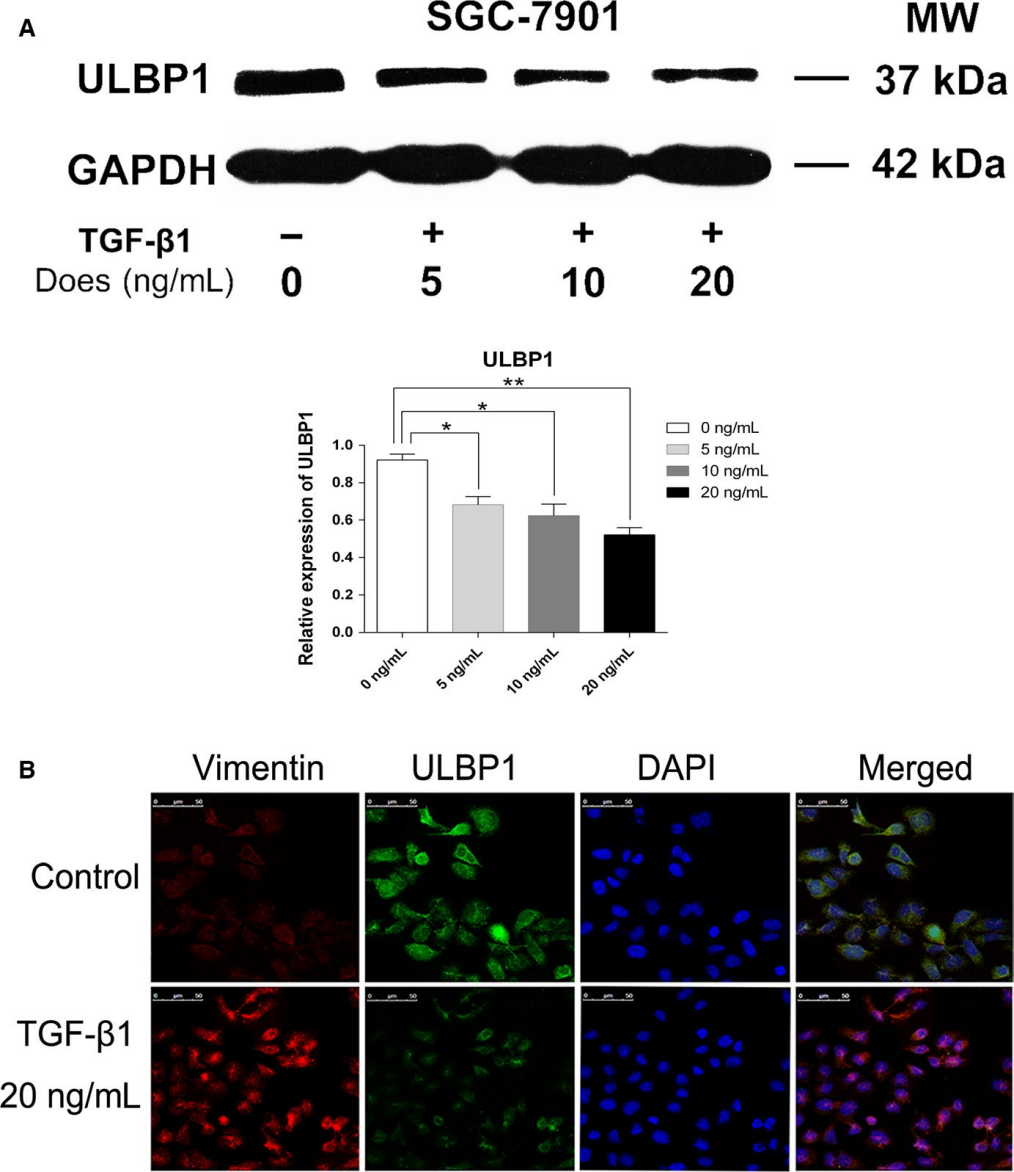
ULBP1 expression in gastric SGC7901 cells. A, ULBP1 protein expression in SGC7901 cells treated with TGF‐β1 (5, 10, and 20 ng/mL) for 72 h. B, Epithelial‐mesenchymal transition and expression of ULBP1 on SGC7901 cells treated with TGF‐β1 (20 ng/mL) for 72 h. Scale bar = 50 μm. *P < .05, **P < .01

TGF‐β1 is considered to be a prototypical cytokine inducer of EMT. The above results showed that ULBP1 surface expression was downregulated on M^+^CTCs. To identify the association between EMT and the expression of ULBP1, we investigated whether the expression of ULBP1 was modulated during the stimulation of EMT with exogenous TGF‐β1 on gastric SGC7901 cells in vitro. As shown in Figure 4A, after treatment with exogenous TGF‐β1 (5, 10, and 20 ng/mL) for 72 hours, a decrease in EpCAM expression, together with an increase in Vimentin protein levels, was observed on SGC7901 cells in a dose‐dependent manner (Figure 4A). We then performed immunofluorescence at 72 hours posttreatment with 20 ng/mL TGF‐β1, and EMT phenotype was observed in the SGC7901 cells (Figure 4B). Similarly, ULBP1 expression was also measured by western blotting and immunofluorescence, and significantly decreased levels of ULBP1 were detected on SGC7901 cells (Figure 5A,B). These results indicated that EMT might be involved in regulating the expression of ULBP1.

## 4 DISCUSSION

In previous decades, efforts have been made to develop technologies for distinguishing CTCs from the billions of nonmalignant blood cells present in the blood.^32^, ^33^, ^34^, ^35^ At present, the isolation and enumeration of CTCs from peripheral blood is considered to be a potential strategy for assessing disease progression and response to therapies, due to its numerous advantages.^36^, ^37^, ^38^, ^39^ Currently, methods of CTC isolation are mostly performed according to cell biological properties, physical properties, or a combination of both.^40^, ^41^ Other methods have also been developed by incorporating multiple principles to achieve optimal cell isolation.^42^ In the current study, we used a negative enrichment technique based on magnetic separation and gradient centrifugation to capture CTCs in peripheral blood samples from gastric cancer patients.^43^, ^44^ Unlike other negative enrichment methods, these magnetic separation and gradient centrifugation methods could deplete WBCs effectively, but not isolate CTCs directly from the millions of remaining cells, which resulted in the detection of nonhypotonic, damaged CTCs, regardless of the physical and biological properties of the CTCs. Meanwhile, to avoid false positive detection of CTCs, we identified CTCs with an immuno‐fluorescence in situ hybridization (im‐FISH) method based on chromosome 8 or 17 ploidy and CD45 expression. As shown in Figure 1, the CTCs exhibited negative membrane staining of CD45, and expressed multiple copies of chromosome 8 or 17 (≥3 green or yellow fluorescent dots), while the WBCs were identified as positive for CD45 (red fluorescence of the cell membrane) and exhibited diploid expression of chromosome 8 or 17 (two green or yellow fluorescence dots). Based on the enumeration of chromosome 8 or 17 and the expression of CD45, CTCs could be easily differentiated from the thousands of other cells. Compared with a previous CTC detection strategy based on the phenotypic characteristics of the CTCs,^45^, ^46^ the method we used in this study was based on karyotyping of the CTCs, which facilitated straightforward identification of nonhematopoietic heteroploid CTCs, regardless of CTC phenotypic changes, such as downregulated or absent expression of EpCAM or other tumor cell surface markers.^47^, ^48^, ^49^ Although the detection rate in our present study was not as high as the phenotypic methods reported previously, we believe that our methods achieved a higher accuracy rate.

Epithelial‐mesenchymal transition has been established to involve the acquisition of a mesenchymal phenotype by epithelial cells. Studies from various research papers have revealed that EMT is a commonly occurring phenomenon during the metastasis of CTCs in various cancers.^50^, ^51^, ^52^, ^53^ In this study, we detected three subtypes of CTCs, namely E^+^CTCs, E^+^/M^+^CTCs, and M^+^CTCs, and found that the positive rate and count range of each subpopulation differed in each CTC‐positive patients. Additionally, the patients with more CTCs seemed to exhibit higher counts of E^+^/M^+^CTCs and M^+^CTCs. Researchers have documented that the phenotypic plasticity of CTCs is a bidirectional conversion among epithelial, mesenchymal, and hybrid epithelial/mesenchymal phenotypes, and that CTCs might successively undergo a process of EMT and MET in metastasis and the formation of secondary tumors.^54^, ^55^, ^56^ Although the case number in our study was small, and we are unsure which subpopulation of CTCs resulted from EMT or MET, our results suggested that EMT may be a common process that CTCs must undergo in the metastasis of gastric cancer. In addition, we were unable to perform an effective statistical analysis on the relationship between CTC EMT phenotype and the clinicopathological features of patients due to the small number of patients enrolled, and thus we intend to include more cases in a future study.

Immune escape and suppression are important phenomena and have long been proposed to constitute critical steps in both tumor formation and progression.^57^, ^58^, ^59^ Theoretically, cancer cells that exit the primary tumor tissue, and thus leave the protection of the immunosuppressive tumor microenvironment, are more vulnerable to attack by immune effector cells.^60^, ^61^ Therefore, the survival of CTCs might be a vulnerable aspect of malignant tumor metastasis, and thus the mechanism enabling CTCs to evade immune effector cell killing may be an important target for prospective cancer therapy research. Studies have shown that tumor cells may shed or otherwise restrict the presentation of NK‐cell receptor D (NKG2D) ligands, such as MICA, MICB, and ULBPs, involved in their recognition by NK cells or cytotoxic T lymphocytes (CTLs), or downregulate the expression of other factors that promote the activation of tumor‐specific immune responses.^62^, ^63^, ^64^, ^65^, ^66^, ^67^ Furthermore, it is established that tumor cells that undergo EMT acquire phenotypic changes, involving the upregulation and downregulation of molecules. Increasing studies have also revealed that CTCs undergo EMT during the process of metastasis.^68^, ^69^, ^70^ However, it remains unclear whether EMT of CTCs is accompanied by a change in ULBP1 expression. Therefore, the present study detected the cell‐surface expression of EMT markers and ULBP1 on CTCs, and found that increased Vimentin and decreased EpCAM expression was accompanied by decreased ULBP1 expression. These results indicated that gastric CTCs might also shed or otherwise restrict the presentation of ULBP1 to escape immune effector killing. Moreover, the alteration of Vimentin and EpCAM expression on CTCs also indicated that EMT might be involved in the regulation of ULBP1 and in the immune evasion of CTCs. To build on previous studies, in which EMT induced a consistent upregulation of NKG2D ligand expression in colorectal carcinoma cell lines,^71^ we performed this detection of EMT in CTCs. Recently, several studies also showed mesenchymal features in lung or breast cancer models was correlated with resistance to NK cells or cytotoxic T lymphocytes attacks.^72^, ^73^, ^74^, ^75^ However, to the best of our knowledge, this is the first study to evaluate the relationship between EMT and ULBP1 expression in gastric CTCs. In addition, we acknowledge that other NKG2D ligands, such as ULBP2‐6, MICA, and MICB, and other markers of EMT, such as E‐cadherin, Twist, and Snail, might also be involved in the interaction of NK cells with tumor cells.^76^, ^77^ To further confirm the role of EMT, as well as the relationship between ULBP1 expression and EMT in the immune evasion of CTCs, functional studies introducing NK cells into a culture system of gastric cancer cell lines, and studies expanding the range of molecules detected on gastric CTCs, are now warranted.

To investigate the correlation between EMT and ULBP1, we successfully induced EMT in the gastric cancer cell line SGC7901 with TGF‐β1 at concentrations of 5, 10, and 20 ng/mL in the current study, and the results showed that EMT markers, including EpCAM and Vimentin, as well as ULBP1, underwent significant changes in expression dependent on TGF‐β1 dosage (Figures 4 and 5). Although the concentrations of TGF‐β1 and the conditions of SGC7901 cell culture in vitro were not completely equivalent to the environment of gastric CTCs in the bloodstream, these results indicated a relationship between EMT and the immune evasion capacity of CTCs, and suggested a possible mechanism regarding CTC immune evasion, where CTCs may shed or restrict the presentation of ULBP1. It has been established that TGF‐β1 is not only a powerful extracellular EMT inducer, but also a crucial cytokine in the regulation of all aspects of the immune response, with a vital role in regulating responses mediated by almost all innate and adaptive immune cells.^78^, ^79^, ^80^ Our results indirectly identified a potential role of TGF‐β1 in the immune evasion of CTCs. Recently, platelets have been reported to promote and/or maintain the state of EMT in CTCs through platelet‐secreted TGF‐β.^81^, ^82^ Moreover, studies have reported that platelets could promote the survival of CTCs in the bloodstream by conferring resistance to the shear stress and to attack from NK cells.^83^, ^84^ However, it is unclear whether downregulation of ULBP1 via EMT‐induced platelet‐secreted TGF‐β is a way in which platelets protect CTCs from immune effector killing. This may be vital in understanding the immune evasion capacity of CTCs and requires further study.

Overall, in this proof‐of‐principle study, we demonstrated that gastric CTCs in peripheral blood exhibited obvious EMT characteristics, as well as a downregulation of ULBP1, with TGF‐β1‐induced EMT accompanied by this downregulation of ULBP1 in vitro. Considering these results, we conclude that EMT of gastric CTCs might be involved in the immune evasion of CTCs through shedding or restricting the expression of ULBP1 during the process of metastasis. Further studies involving expanded NKG2D ligand expression on CTCs, its effect on immune evasion of CTCs and the possible regulation mechanism should be performed in the future to confirm this conclusion.

## CONFLICT OF INTEREST

The authors declare that there was no conflict of interest regarding the publication of this paper.

## AUTHOR CONTRIBUTIONS

Baoguang Hu was involved in conceptualization and project administration. Xiaokun Tian was involved in manuscript writing. Yanbin Li and Chunyang Liu were involved in in vitro experiments. Yuming Li was involved in critical revision and final approval of the manuscript. Zhaodong Han, Tao Yang, and Jiajia An were involved in collection and analysis of clinical samples. Lingqun Kong was involved in collection of the clinical data.

## References

[1] Siegel RL , Miller KD , Jemal A . Cancer statistics, 2019. CA Cancer J Clin. 2019;69:7‐34. 30620402 10.3322/caac.21551

[2] Miller KD , Nogueira L , Mariotto AB , et al. Cancer treatment and survivorship statistics, 2019. CA Cancer J Clin. 2019;69(5):363‐385. 31184787 10.3322/caac.21565

[3] Bray F , Ferlay J , Soerjomataram I , Siegel RL , Torre LA , Jemal A . Global cancer statistics 2018: GLOBOCAN estimates of incidence and mortality worldwide for 36 cancers in 185 countries. CA Cancer J Clin. 2018;68:394‐424. 30207593 10.3322/caac.21492

[4] Mpallas KD , Lagopoulos VI , Kamparoudis AG . Prognostic significance of solitary lymphnode metastasis and micrometastasis in gastric cancer. Front Surg. 2018;5:63. 30406109 10.3389/fsurg.2018.00063 PMC6200848

[5] Chaffer CL , Weinberg RA . A perspective on cancer cell metastasis. Science. 2011;331:1559‐1564. 21436443 10.1126/science.1203543

[6] Li T‐T , Liu H , Yu J , Shi G‐Y , Zhao L‐Y , Li G‐X . Prognostic and predictive blood biomarkers in gastric cancer and the potential application of circulating tumor cells. World J Gastroenterol. 2018;24:2236‐2246. 29881233 10.3748/wjg.v24.i21.2236 PMC5989238

[7] Hu Y , Yu X , Xu G , Liu S . Metastasis: an early event in cancer progression. J Cancer Res Clin Oncol. 2017;143(5):745‐757. 27686824 10.1007/s00432-016-2279-0 PMC11819082

[8] Gkountela S , Szczerba B , Donato C , Aceto N . Recent advances in the biology of human circulating tumour cells and metastasis. ESMO Open. 2016;1:e000078. 27843628 10.1136/esmoopen-2016-000078 PMC5070257

[9] Paoletti C , Hayes DF . Circulating tumor cells. Adv Exp Med Biol. 2016;882:235‐258. 26987538 10.1007/978-3-319-22909-6_10

[10] Kolostova K , Spicka J , Matkowski R , Bobek V . Isolation, primary culture, morphological and molecular characterization of circulating tumor cells in gynecological cancers. Am J Transl Res. 2015;7:1203‐1213. 26328005 PMC4548313

[11] Pantel K , Alix‐Panabieres C . Functional studies on viable circulating tumor cells. Clin Chem. 2016;62(2):328‐334. 26637479 10.1373/clinchem.2015.242537

[12] Marcuello M , Vymetalkova V , Neves RPL , et al. Circulating biomarkers for early detection and clinical management of colorectal cancer. Mol Aspects Med. 2019;69:107‐122. 31189073 10.1016/j.mam.2019.06.002

[13] Galanzha EI , Menyaev YA , Yadem AC , et al. In vivo liquid biopsy using Cytophone platform for photoacoustic detection of circulating tumor cells in patients with melanoma. Sci Transl Med. 2019;11:eaat5857. 31189720 10.1126/scitranslmed.aat5857 PMC9235419

[14] Buscail E , Chiche L , Laurent C , et al. Tumor‐proximal liquid biopsy to improve diagnostic and prognostic performances of circulating tumor cells. Mol Oncol. 2019;13(9):1811‐1826. 31216108 10.1002/1878-0261.12534 PMC6717761

[15] Wang H , Stoecklein NH , Lin PP , Gires O . Circulating and disseminated tumor cells: diagnostic tools and therapeutic targets in motion. Oncotarget. 2017;8:1884‐1912. 27683128 10.18632/oncotarget.12242 PMC5352105

[16] Friedlander TW , Welty C , Anantharaman A , et al. Identification and characterization of circulating tumor cells in men who have undergone prostatectomy for clinically localized, high risk prostate cancer. J Urol. 2019;202(4):732‐741. 31216253 10.1097/JU.0000000000000393

[17] Yao Y , Jing J . [Detection and clinical significance of circulating tumor cells in gastric cancer]. Zhonghua Wei Chang Wai Ke Za Zhi. 2016;19:1077‐1080. 27680080

[18] Zhang Y , Wang F , Ning N , et al. Patterns of circulating tumor cells identified by CEP8, CK and CD45 in pancreatic cancer. Int J Cancer. 2015;136:1228‐1233. 25042121 10.1002/ijc.29070

[19] Zhou J , Ma X , Bi F , Liu M . Clinical significance of circulating tumor cells in gastric cancer patients. Oncotarget. 2017;8:25713‐25720. 28147337 10.18632/oncotarget.14879 PMC5421964

[20] Okabe H , Tsunoda S , Hosogi H , et al. Circulating tumor cells as an independent predictor of survival in advanced gastric cancer. Ann Surg Oncol. 2015;22:3954‐3961. 25777087 10.1245/s10434-015-4483-6

[21] Li TT , Liu H , Li FP , et al. Evaluation of epithelial‐mesenchymal transitioned circulating tumor cells in patients with resectable gastric cancer: relevance to therapy response. World J Gastroenterol. 2015;21:13259‐13267. 26715808 10.3748/wjg.v21.i47.13259 PMC4679757

[22] Yuan D , Xia H , Zhang Y , et al. P‐Akt/miR200 signaling regulates epithelial‐mesenchymal transition, migration and invasion in circulating gastric tumor cells. Int J Oncol. 2014;45:2430‐2438. 25200917 10.3892/ijo.2014.2644

[23] Zhang ZY , Ge HY . Micrometastasis in gastric cancer. Cancer Lett. 2013;336:34‐45. 23624301 10.1016/j.canlet.2013.04.021

[24] Nicolazzo C , Gradilone A , Carpino G , Gazzaniga P , Raimondi C . Molecular characterization of circulating tumor cells to study cancer immunoevasion. Methods Mol Biol. 2019;1884:247‐258. 30465208 10.1007/978-1-4939-8885-3_17

[25] Chistiakov DA , Chekhonin VP . Circulating tumor cells and their advances to promote cancer metastasis and relapse, with focus on glioblastoma multiforme. Exp Mol Pathol. 2018;105:166‐174. 30028961 10.1016/j.yexmp.2018.07.007

[26] Yue J , Sun H , Liu S , et al. Downregulation of NDR1 contributes to metastasis of prostate cancer cells via activating epithelial‐mesenchymal transition. Cancer Med. 2018;7(7):3200‐3212. 10.1002/cam4.1532 PMC6051198 29733518

[27] Jolly MK , Tripathi SC , Somarelli JA , Hanash SM , Levine H . Epithelial/mesenchymal plasticity: how have quantitative mathematical models helped improve our understanding? Mol Oncol. 2017;11:739‐754. 28548388 10.1002/1878-0261.12084 PMC5496493

[28] Shen Q , Shen LS , Chen Q , Zhou JY , Zhou JY . The value of circulating tumor cells detected by chromosome centromere probe identification in diagnosis of non‐small cell lung cancer. Zhonghua Jie He He Hu Xi Za Zhi. 2018;41:772‐777. 30347548 10.3760/cma.j.issn.1001-0939.2018.10.005

[29] Li Y , Ma G , Zhao P , et al. Improvement of sensitive and specific detection of circulating tumor cells using negative enrichment and immunostaining‐FISH. Clin Chim Acta. 2018;485:95‐102. 29940145 10.1016/j.cca.2018.06.034

[30] Ning N , Zhan T , Zhang Y , et al. Improvement of specific detection of circulating tumor cells using combined CD45 staining and fluorescence in situ hybridization. Clin Chim Acta. 2014;433:69‐75. 24607330 10.1016/j.cca.2014.02.019

[31] Ge F , Zhang H , Wang DD , Li L , Lin PP . Enhanced detection and comprehensive in situ phenotypic characterization of circulating and disseminated heteroploid epithelial and glioma tumor cells. Oncotarget. 2015;6:27049‐27064. 26267323 10.18632/oncotarget.4819 PMC4694973

[32] Shen Z , Wu A , Chen X . Current detection technologies for circulating tumor cells. Chem Soc Rev. 2017;46:2038‐2056. 28393954 10.1039/c6cs00803h PMC5598784

[33] Satelli A , Batth I , Brownlee Z , et al. EMT circulating tumor cells detected by cell‐surface vimentin are associated with prostate cancer progression. Oncotarget. 2017;8:49329‐49337. 28521303 10.18632/oncotarget.17632 PMC5564771

[34] Liu Y‐K , Hu B‐S , Li Z‐L , He XU , Li Y , Lu L‐G . An improved strategy to detect the epithelial‐mesenchymal transition process in circulating tumor cells in hepatocellular carcinoma patients. Hepatol Int. 2016;10:640‐646. 27115761 10.1007/s12072-016-9732-7

[35] Watanabe M , Uehara Y , Yamashita N , et al. Multicolor detection of rare tumor cells in blood using a novel flow cytometry‐based system. Cytometry A. 2014;85:206‐213. 24327318 10.1002/cyto.a.22422

[36] Woo D , Yu M . Circulating tumor cells as "liquid biopsies" to understand cancer metastasis. Transl Res. 2018;201:128‐135. 30075099 10.1016/j.trsl.2018.07.003 PMC6177282

[37] Zhang J , Chen K , Fan ZH . Circulating tumor cell isolation and analysis. Adv Clin Chem. 2016;75:1‐31. 27346614 10.1016/bs.acc.2016.03.003 PMC5123699

[38] Kaifi JT , Li G , Clawson G , Kimchi ET , Staveley‐O'Carroll KF . Perioperative circulating tumor cell detection: current perspectives. Cancer Biol Ther. 2016;17:859‐869. 27045201 10.1080/15384047.2016.1167296 PMC5004694

[39] Joosse SA , Gorges TM , Pantel K . Biology, detection, and clinical implications of circulating tumor cells. EMBO Mol Med. 2015;7:1‐11. 25398926 10.15252/emmm.201303698 PMC4309663

[40] Yanagita M , Luke JJ , Hodi FS , Janne PA , Paweletz CP . Isolation and characterization of circulating melanoma cells by size filtration and fluorescent in‐situ hybridization. Melanoma Res. 2018;28:89‐95. 29406397 10.1097/CMR.0000000000000431

[41] Turetta M , Ben FD , Brisotto G , et al. Emerging technologies for cancer research: towards personalized medicine with microfluidic platforms and 3D tumor models. Curr Med Chem. 2018;25:4616‐4637. 29874987 10.2174/0929867325666180605122633 PMC6302350

[42] Harouaka R , Kang Z , Zheng SY , Cao L . Circulating tumor cells: advances in isolation and analysis, and challenges for clinical applications. Pharmacol Ther. 2014;141:209‐221. 24134902 10.1016/j.pharmthera.2013.10.004 PMC3947247

[43] Chen YY , Xu GB . Effect of circulating tumor cells combined with negative enrichment and CD45‐FISH identification in diagnosis, therapy monitoring and prognosis of primary lung cancer. Med Oncol. 2014;31:240. 25361883 10.1007/s12032-014-0240-0

[44] Kanwar N , Done SJ . Negative enrichment and isolation of circulating tumor cells for whole genome amplification. Methods Mol Biol. 2017;1634:143‐152. 28819847 10.1007/978-1-4939-7144-2_11

[45] Gabriel MT , Calleja LR , Chalopin A , Ory B , Heymann D . Circulating tumor cells: a review of non‐EpCAM‐based approaches for cell enrichment and isolation. Clin Chem. 2016;62:571‐581. 26896446 10.1373/clinchem.2015.249706

[46] Benini S , Gamberi G , Cocchi S , et al. Detection of circulating tumor cells in liquid biopsy from Ewing sarcoma patients. Cancer Manag Res. 2018;10:49‐60. 29386915 10.2147/CMAR.S141623 PMC5765973

[47] Mohme M , Riethdorf S , Pantel K . Circulating and disseminated tumour cells – mechanisms of immune surveillance and escape. Nat Rev Clin Oncol. 2017;14:155‐167. 27644321 10.1038/nrclinonc.2016.144

[48] Hyun K‐A , Koo G‐B , Han H , et al. Epithelial‐to‐mesenchymal transition leads to loss of EpCAM and different physical properties in circulating tumor cells from metastatic breast cancer. Oncotarget. 2016;7:24677‐24687. 27013581 10.18632/oncotarget.8250 PMC5029733

[49] Gorges TM , Tinhofer I , Drosch M , et al. Circulating tumour cells escape from EpCAM‐based detection due to epithelial‐to‐mesenchymal transition. BMC Cancer. 2012;12:178. 22591372 10.1186/1471-2407-12-178 PMC3502112

[50] Manjunath Y , Upparahalli SV , Avella DM , et al. PD‐L1 expression with epithelial mesenchymal transition of circulating tumor cells is associated with poor survival in curatively resected non‐small cell lung cancer. Cancers. 2019;11(6):806. 10.3390/cancers11060806 PMC6628040 31212653

[51] Pore M , Meijer C , de Bock GH , et al. Cancer stem cells, epithelial to mesenchymal markers, and circulating tumor cells in small cell lung cancer. Clin Lung Cancer. 2016;17:535‐542. 27363902 10.1016/j.cllc.2016.05.015

[52] Hamilton G , Hochmair M , Rath B , Klameth L , Zeillinger R . Small cell lung cancer: circulating tumor cells of extended stage patients express a mesenchymal‐epithelial transition phenotype. Cell Adh Migr. 2016;10:360‐367. 26919626 10.1080/19336918.2016.1155019 PMC4986707

[53] Mego M , Cierna Z , Janega P , et al. Relationship between circulating tumor cells and epithelial to mesenchymal transition in early breast cancer. BMC Cancer. 2015;15:533. 26194471 10.1186/s12885-015-1548-7 PMC4509773

[54] Garg M . Epithelial, mesenchymal and hybrid epithelial/mesenchymal phenotypes and their clinical relevance in cancer metastasis. Expert Rev Mol Med. 2017;19:e3. 28322181 10.1017/erm.2017.6

[55] Alix‐Panabieres C , Mader S , Pantel K . Epithelial‐mesenchymal plasticity in circulating tumor cells. J Mol Med (Berl). 2017;95:133‐142. 28013389 10.1007/s00109-016-1500-6

[56] Wu S , Liu S , Liu Z , et al. Classification of circulating tumor cells by epithelial‐mesenchymal transition markers. PLoS ONE. 2015;10:e0123976. 25909322 10.1371/journal.pone.0123976 PMC4409386

[57] Leone K , Poggiana C , Zamarchi R . The interplay between circulating tumor cells and the immune system: from immune escape to cancer immunotherapy. Diagnostics. 2018;8(3):59. 10.3390/diagnostics8030059 PMC6164896 30200242

[58] Parcesepe P , Giordano G , Laudanna C , Febbraro A , Pancione M . Cancer‐associated immune resistance and evasion of immune surveillance in colorectal cancer. Gastroenterol Res Pract. 2016;2016:6261721. 27006653 10.1155/2016/6261721 PMC4781955

[59] Prendergast GC . Immune escape as a fundamental trait of cancer: focus on IDO. Oncogene. 2008;27:3889‐3900. 18317452 10.1038/onc.2008.35

[60] Dasgupta A , Lim AR , Ghajar CM . Circulating and disseminated tumor cells: harbingers or initiators of metastasis? Mol Oncol. 2017;11:40‐61. 28085223 10.1002/1878-0261.12022 PMC5423226

[61] Noman MZ , Messai Y , Muret J , Hasmim M , Chouaib S . Crosstalk between CTC, immune system and hypoxic tumor microenvironment. Cancer Microenviron. 2014;7:153‐160. 25337680 10.1007/s12307-014-0157-3 PMC4275540

[62] Wang D , Gu X , Liu X , et al. "Acquired" NKG2D ligand stimulates NK cell‐mediated tumor immunosurveillance. J Immunother. 2019;42:189‐196. 31145234 10.1097/CJI.0000000000000276

[63] Frazao A , Rethacker L , Messaoudene M , et al. NKG2D/NKG2‐ligand pathway offers new opportunities in cancer treatment. Front Immunol. 2019;10:661. 30984204 10.3389/fimmu.2019.00661 PMC6449444

[64] Dhar P , Wu JD . NKG2D and its ligands in cancer. Curr Opin Immunol. 2018;51:55‐61. 29525346 10.1016/j.coi.2018.02.004 PMC6145810

[65] Liu X , Sun M , Yu S , et al. Potential therapeutic strategy for gastric cancer peritoneal metastasis by NKG2D ligands‐specific T cells. Onco Targets Ther. 2015;8:3095‐3104. 26543378 10.2147/OTT.S91122 PMC4622417

[66] Vantourout P , Willcox C , Turner A , et al. Immunological visibility: posttranscriptional regulation of human NKG2D ligands by the EGF receptor pathway. Sci Transl Med. 2014;6:231ra249. 10.1126/scitranslmed.3007579 PMC3998197 24718859

[67] Shi P , Yin T , Zhou F , Cui P , Gou S , Wang C . Valproic acid sensitizes pancreatic cancer cells to natural killer cell‐mediated lysis by upregulating MICA and MICB via the PI3K/Akt signaling pathway. BMC Cancer. 2014;14:370. 24885711 10.1186/1471-2407-14-370 PMC4076062

[68] Liu X , Li J , Cadilha BL , et al. Epithelial‐type systemic breast carcinoma cells with a restricted mesenchymal transition are a major source of metastasis. Sci Adv. 2019;5:eaav4275. 31223646 10.1126/sciadv.aav4275 PMC6584608

[69] Zhao R , Cai Z , Li S , et al. Expression and clinical relevance of epithelial and mesenchymal markers in circulating tumor cells from colorectal cancer. Oncotarget. 2017;8:9293‐9302. 28030836 10.18632/oncotarget.14065 PMC5354732

[70] Masuda T , Hayashi N , Iguchi T , et al. Clinical and biological significance of circulating tumor cells in cancer. Mol Oncol. 2016;10:408‐417. 26899533 10.1016/j.molonc.2016.01.010 PMC5528976

[71] López‐Soto A , Huergo‐Zapico L , Galván JA , et al. Epithelial‐mesenchymal transition induces an antitumor immune response mediated by NKG2D receptor. J Immunol. 2013;190:4408‐4419. 23509364 10.4049/jimmunol.1202950

[72] Terry S , Buart S , Tan TZ , et al. Acquisition of tumor cell phenotypic diversity along the EMT spectrum under hypoxic pressure: consequences on susceptibility to cell‐mediated cytotoxicity. Cancer Res. 2017;6:e1271858. 10.1080/2162402X.2016.1271858 PMC5353930 28344883

[73] Al Absi A , Wurzer H , Guerin C , et al. Actin cytoskeleton remodeling drives breast cancer cell escape from natural killer–mediated cytotoxicity. Cancer Res. 2018;78(19):5631‐5643. 30104240 10.1158/0008-5472.CAN-18-0441

[74] MacFawn I , Wilson H , Selth LA , et al. Grainyhead‐like‐2 confers NK‐sensitivity through interactions with epigenetic modifiers. Mol Immunol. 2019;105:137‐149. 30508726 10.1016/j.molimm.2018.11.006 PMC6585439

[75] Terry S , Abdou A , Engelsen AST , et al. Actin cytoskeleton remodeling drives breast cancer cell escape from natural killer–mediated cytotoxicity. Cancer Res. 2018;78(19):5631‐5643. 30104240 10.1158/0008-5472.CAN-18-0441

[76] Duan S , Guo W , Xu Z , et al. Natural killer group 2D receptor and its ligands in cancer immune escape. Mol Cancer. 2019;18:29. 30813924 10.1186/s12943-019-0956-8 PMC6391774

[77] Cosman D , Müllberg J , Sutherland CL , et al. ULBPs, novel MHC class I‐related molecules, bind to CMV glycoprotein UL16 and stimulate NK cytotoxicity through the NKG2D receptor. Immunity. 2001;14:123‐133. 11239445 10.1016/s1074-7613(01)00095-4

[78] Kelly A , Houston SA , Sherwood E , Casulli J , Travis MA . Regulation of innate and adaptive immunity by TGFbeta. Adv Immunol. 2017;134:137‐233. 28413021 10.1016/bs.ai.2017.01.001

[79] Saitoh M . Epithelial‐mesenchymal transition is regulated at post‐transcriptional levels by transforming growth factor‐beta signaling during tumor progression. Cancer Sci. 2015;106:481‐488. 25664423 10.1111/cas.12630 PMC4452147

[80] Katsuno Y , Lamouille S , Derynck R . TGF‐beta signaling and epithelial‐mesenchymal transition in cancer progression. Curr Opin Oncol. 2013;25:76‐84. 23197193 10.1097/CCO.0b013e32835b6371

[81] Lou XL , Sun J , Gong SQ , et al. Interaction between circulating cancer cells and platelets: clinical implication. Chin J Cancer Res. 2015;27:450‐460. 26543331 10.3978/j.issn.1000-9604.2015.04.10 PMC4626816

[82] Lou X‐L , Deng J , Deng H , et al. Aspirin inhibit platelet‐induced epithelial‐to‐mesenchymal transition of circulating tumor cells (Review). Biomed Rep. 2014;2:331‐334. 24748969 10.3892/br.2014.242 PMC3990215

[83] Lu Y , Lian S , Ye Y , et al. S‐Nitrosocaptopril prevents cancer metastasis in vivo by creating the hostile bloodstream microenvironment against circulating tumor cells. Pharmacol Res. 2019;139:535‐549. 30366102 10.1016/j.phrs.2018.10.020

[84] Leblanc R , Peyruchaud O . Metastasis: new functional implications of platelets and megakaryocytes. Blood. 2016;128:24‐31. 27154188 10.1182/blood-2016-01-636399

